# Adaptive Epigenetic Differentiation between Upland and Lowland Rice Ecotypes Revealed by Methylation-Sensitive Amplified Polymorphism

**DOI:** 10.1371/journal.pone.0157810

**Published:** 2016-07-05

**Authors:** Hui Xia, Weixia Huang, Jie Xiong, Tao Tao, Xiaoguo Zheng, Haibin Wei, Yunxia Yue, Liang Chen, Lijun Luo

**Affiliations:** 1 Shanghai Agrobiological Gene Center, Shanghai, China; 2 College of Plant Sciences & Technology, Huazhong Agricultural University, Wuhan, China; National Institute of Plant Genome Research, INDIA

## Abstract

The stress-induced epimutations could be inherited over generations and play important roles in plant adaption to stressful environments. Upland rice has been domesticated in water-limited environments for thousands of years and accumulated drought-induced epimutations of DNA methylation, making it epigenetically differentiated from lowland rice. To study the epigenetic differentiation between upland and lowland rice ecotypes on their drought-resistances, the epigenetic variation was investigated in 180 rice landraces under both normal and osmotic conditions *via* methylation-sensitive amplified polymorphism (MSAP) technique. Great alterations (52.9~54.3% of total individual-locus combinations) of DNA methylation are recorded when rice encountering the osmotic stress. Although the general level of epigenetic differentiation was very low, considerable level of ΦST (0.134~0.187) was detected on the highly divergent epiloci (HDE). The HDE detected in normal condition tended to stay at low levels in upland rice, particularly the ones de-methylated in responses to osmotic stress. Three out of four selected HDE genes differentially expressed between upland and lowland rice under normal or stressed conditions. Moreover, once a gene at HDE was up-/down-regulated in responses to the osmotic stress, its expression under the normal condition was higher/lower in upland rice. This result suggested expressions of genes at the HDE in upland rice might be more adaptive to the osmotic stress. The epigenetic divergence and its influence on the gene expression should contribute to the higher drought-resistance in upland rice as it is domesticated in the water-limited environment.

## Introduction

How plants adapt to the stressful environment is a fundamental question in the evolutionary biology of plants. Apart from the genetic mechanisms, epigenetic processes are considered having evolutionary significances in plant adaption to the stressful environment [1–3], including the DNA methylation [4–6]. Many studies have shown that the DNA methylation in plants is sensitive to various abiotic stressors, such as salt [7], nitrogen-deficiency [8], temperature [9–11], osmotic [12], and drought [13, 14], making the plant a rapid response to the stress[15–17].The epimutation of DNA methylation could be generated at a higher rate than equivalent genetic mutations [18], particularly in the stressful environment. Some of the stress-induced epimutation could be inherited over generations [19–22], forming novel epigenetic variants [23]. As the epimutation of DNA methylation contributes to heritable phenotypes [4, 15, 24], the stress-induced epigenetic variants could be the target of natural selection [8, 25]. The transgenerational inherited epimutations in the plant populations from different habitats promotes adaptive epigenetic divergence among them [5, 26]. Although many studies have described the role played by the DNA methylation in plant populations of wild species adapting to different ecosystems [27–30], the adaptive epigenetic divergence among ecotypes of a crop under the human selection (domestication) is still rare. Given the important agricultural traits are always promoted during the adaption of crop ecotypes to different agro-ecosystems [31], the adaptive epigenetic divergence of DNA methylation among crop ecotypes and its underlying mechanism should be studied with emphasis.

Asia cultivated rice (*Oryza sativa* L.) is one of the most important cereal crops. During the process of domestication, upland and lowland rice have adapted to different agricultural ecosystems of contrasting soil-water conditions [32]. Upland rice is always planted in the unbunded fields with good drainage of soil. On the contrary, lowland rice is commonly planted in fields with well water maintaining and irrigation conditions. Consequently, upland rice encounters higher risks of drought and has acquired higher drought-resistances [32, 33]. However, only very limited genetic divergence has been detected between upland and lowland rice which cannot fully explain their differences on the drought-resistance [33, 34].On the contrary, great alterations of DNA methylation have been detected when rice encountering the drought stress [7, 13, 14] and some of these drought-induced epimutations could be transgenerational inherited to next few generations [35]. All these findings raise questions that whether the stress-induced epimutation of DNA methylation could lead to adaptive epigenetic divergence between upland and lowland rice ecotypes and whether the epigenetic divergence is associated with the drought-resistance?

The osmotic stress (also called hypertonic dehydration) always occurs along with the drought and the osmotic-tolerance is a vital part of the drought-resistance [36]. As the epigenetic status of a plant could be largely influenced by its development [37], it is difficult to fairly investigate the DNA methylation in large amount of rice landraces with varied growth period under field conditions to the drought stress. Therefore, we decided to investigate epigenetic status of rice at the seedling stage to the osmotic stress which could be conducted in well-controlled laboratory conditions. By this design, we can explore the adaptive epigenetic divergence between rice ecotypes on their drought-resistances.

The technique of methylation sensitive amplification polymorphisms (MSAP) is modified from the amplified fragment length polymorphism (AFLP) by using a pair of isoschizomeric restriction enzymes with different sensitivities to site-specific cytosine methylation. The MSAP method has been widely used in studies of ecological and population epigenetics in latest years [29, 38–40]. To explore the evolutionary significance of DNA methylation in rice ecotypes adapting to agro-ecosystems of contrasting soil-water conditions, the adaptive epigenetic divergence and its influence on the gene expression between upland and lowland rice ecotypes are investigated.

## Materials and Methods

### Ethics statement

All the rice materials were planted in the fields of experimental station belonged to Shanghai Academy of Agricultural Sciences to gain the seeds and the laboratory experiment was conducted under indoor conditions. We have granted the permission to conduct these field and laboratory experiments. There were no endangered wild species in the experimental station and the laboratory experiment had no negative impacts to the environment.

### Plant materials

180 plant materials, including 71 *japonica-*upland, 59 *japonica*-lowland, 24 *indica*-upland, and 26 *indica*-lowland landraces from four provinces of China were involved in this study to investigate the epigenetic variance and differentiation between upland rice and lowland rice ecotypes (Table 1).

**Table 1:** Rice landraces and their basic information.

Region	Group (subspeices-ecotype)			
	*Japonica*-upland	*Japonica*-lowland	*Indica*-upland	*Indica*-lowland
Hebei	21 (Pop3)	19 (Pop7)	0	0
Jiangsu	14 (Pop4)	23 (Pop8)	0	0
Guangxi	15 (Pop1)	0	24 (Pop5)	26 (Pop9)
Guizhou	21 (Pop2)	17 (Pop6)	0	0
Overall	71	59	24	26

### Normal and osmotic treatment

Seeds of the 180rice landraces were germinated on the 48-well plate and growing in agrowth chamber (14 hours of daytime at 30°C and 10 hours of night at 20°C with 70% relative humidity).One rice landrace had two plates with 24 individuals per plate. After 20 days of growing in the normal nutrient solution, one plate was treated with 20% PEG6000 to simulate the osmotic stress (OS), while the other plate was kept in the normal nutrient solution as control (CK). After treatedin20% PEG6000 for 24hours, three individual seedlings of each landrace in the OS plate were harvested when they showed signs of slight leaf rolling. The relative water content of seedlings under OS condition were similar (p = 0.405) in upland rice (61.8±0.9%) and in lowland rice (62.9±1.0%). At the same time, three individual seedlings of each landrace in the CK plate were also sampled.

### Procedures DNA extraction and MSAP genotyping

Total genomic DNA was extracted following the common cetyltrimethyl ammonium bromide (CTAB) protocol. Three seedlings of each materials were mixed together to include the epigenetic variation within a material. The procedure of MSAP was described in detail in our previous study [35]. 16selective primer combinations were involved in this study (S1 Table). The 5’ end of the selective primer was labeled with fluorescent dyes. The PCR products were then analyzed on ABI 3130XL (Applied Biosystems, USA) using ROX500 as an internal standard. The resulting chromatograms were analyzed by Peakscanner ver. 1.0 and then scored manually. Based on the visualized chromatograms, MSAP bands could be accurately separated and scored (S1 Fig).

Comparisons of the banding patterns of EcoRI/HpaII and EcoRI/MspI reactions results in four conditions of a particular fragment, representing different types of DNA methylation (Table 2).The “0/0” type could be determined as hyper-methylation if it represented other methylation types in the CK or OS condition. To be more cautious, if the proportion of the uninformative type V (scored as “0/0” in both CK and OS conditions) exceeded 10%, this epilocus was excluded in our data set. To test the particular impacts of the methylation type I (un-methylation), II (hemi-methylation), III (fully-methylation), and VI (hyper-methylation), the improved ‘Mixed-Scoring 2’ approach [29] was used to study the epigenetic differentiation between upland and lowland rice ecotypes (Table 2). It generated the final epigenetic data matrix by transforming the four discernible methylation types of each multistate epilocus into separate binary sub-epiloci (S3 Table).

**Table 2:** Four epigenotypes of methylation conditions, their methylation degrees, and the scores of four sub-epiloci transformed by the modified approach of “Mixed Scoring 2”.

Methylation type	Methylation status	Methylation degree	Raw data (H/M)	Mixed scoring (sub-epiloci)			
				I: Non-methylation	II: ^Me^CG / ^HMe^CG	III: ^HMe^CGG	IV: ^Me^CGG
I: Non-methylation	CCGG	0	1/1	1	0	0	0
	GGCC						
II: ^Me^CG or ^HMe^CG	C^me^CGG C^me^CGG	1	0/1	0	1	0	0
	GG^me^CC GGCC						
III: ^HMe^CGG	^me^CCGG	1	1/0	0	0	1	0
	GGCC						
IV: ^Me^CGG	^me^CCGG ^me^C^me^CGG	2	0/0	0	0	0	1
	GGC^me^C GG^me^C^me^C						
V: Genetic mutation	CC***T***G	NA	0/0	NA	NA	NA	NA
	GG***A***C						

### Data analysis

#### DNA methylation status and epigenetic divergence between upland and lowland rice in CK and OS conditions

The DNA methylation level of each epilocus was calculated as the proportion of (Type II + Type II + Type IV)/ (Type I + Type II + Type III + Type IV). The epigenetic diversity was quantified by the percentage of polymorphic loci (*PLP*) and the Shannon’s information index (*H’*). The population structure of the four subspecies-ecotypeswas described by principal coordinates analyses (PCoA) based on the distance matrix calculated by GenAlex ver. 6.43 using the total data and four types of the sub-epiloci (I, II,III, and IV) separately (S2 Table). Epigenetic variation among populations and groups was quantified by the hierarchical analysis of molecular variance (AMOVA) implemented in GenAlex ver. 6.43. The AMOVA were also conducted using the SSR data extracted from our previous study of same plant materials [33]. The ΦST/ΦCT was calculated as the percentage of epigenetic/genetic variance between populations to the total epigenetic variance *via* AMOVA, representing the level of epigenetic/genetic divergence. The pairwise ΦSTs were calculated among the nine populations respectively using the epigenetic data of CK and OS, as well as the ΦCT using the SSR data extracted from our previous study [33]. Mantel tests were conducted among these pairwise epigenetic and genetic matrices.

#### Determination of highly divergent epilocus and its behavior in CK and OS conditions

Highly divergent epilocus (HDE) was defined as the epilocus containing at least one of the four types of sub-epiloci with the ΦST beyond 95% confidence interval (CI) among total sub-epiloci. It was detected respectively in the CK (HDE-CK) and OS (HDE-OS) conditions. Corresponding sub-epilocus of I, II, III, and IV types belonged to the one epilocus were jointly excluded from the neutral epiloci when any one of them was determined as the HDE.

The HDE-CK represents different methylation status between upland and lowland rice in CK. Methylation levels of HDE-CK and the neutral loci were respectively calculated in upland and lowland rice under CK and OS conditions. The HDE-OS represents different methylation status between upland and lowland rice in OS, resulting from the different responses of DNA methylation to the stress. As the methylation degrees were assigned to the four methylation types (I, II, III, and IV) (Table 2), the degree of DNA methylation alterations could be quantified by the difference of methylation degree (DMD) from CK to OS. It was calculated as: methylation degree in OS—methylation degree in CK. The positive/negative value of the DMD mean re-methylation/de-methylation in responses to the osmotic stress at this epilocus. The difference of DMD between upland and lowland rice on HDE-OS was expected to be significantly higher than that on the neutral epiloci. To test these, independent *t*-test were conducted *via* SPSS ver. 15.0.

#### The annotation of genes at epigenetic loci and their expressions

PCR products between 100-400bpfrom five MSAP primer combinations (S3 Table) were recycled from 1.5% agorose gel and directly sent to sequencing *via* Illumina HiSeq 2500. The quality-controlled reads were mapped to the reference genome MSU 7.0. Although there were thousands of sequenced DNA fragments in the PCR products, the one with higher abundance should be resulted from the reaction of the enzymes and selective amplification, forming the stronger signal in the chromatogram. Therefore, only the DNA fragments with depth >200were considered as the potential epilocus scored in this study. We then calculated the molecular length (bp) of these sequenced fragments between the cutting sites of the two restriction enzyme (*EcoRI*: G|AATTC; *HpaII*/*MspI*: C|CGG). If the length of a fragment rigorously matched with the molecular weight of a scored MSAP band (±0.5bp), it was then determined as the corresponding epilocus. To validate the results of direct sequencing from PCR products, four MSAP epiloci (M102, M112, M117, and M124) were recycled from the polyacrylamide gel as they had clear bands near the marker ladder standards (100bp, 150bp, 200bp and 250bp). They were sequenced after cloned to the pEASYR-Blunt Simple Cloning Vector (TRANSGEN BIOTECH Company, China, Product#CB111) (S4 Table).

Expression levels of four HDE genes were then quantified by RT-PCR in5-10 upland and lowland materials in CK and OS conditions using ecotype-preferential epigenotypes with three individual replicates for each material (S5 Table). The *Actin* was used as the reference to calculate the relative expression levels of these genes. Fold difference (FD) between upland and lowland rice was calculated as: expression in upland rice / that in lowland rice while fold change (FC) between CK and OS conditions was calculated as: expression in OS/ that in CK.

## Results

### Methylation status of the four rice groups in CK and OS conditions

There were 1217scoredepiloci on the 16 MSAP primers of which 745 (61.2%) were polymorphic. These polymorphic epiloci yielded 313 informative epiloci ranging from 7~39 for each MSAP primer combinations (S1 and S2 Tables). These 313 informative loci further yielded 1198 and 1188polymorphic sub-epiloci in CK and OS conditions (S2 Table).

There were great variations of methylation levels (from 0~100%) among the 313 raw MSAP loci (S2 Fig) and no significant difference was detected among groups (Table 3). The percentages of polymorphic loci (*PLP*) for the four groups were 79.7–93.5% and 81.4–93.7% respectively in the CK and OS conditions, also with no detectable significance (Table 3). The mean Shannon’s information indices (*H’*) ranged from 0.532~0.541 among the four rice groups in CK while they ranged from 0.528~0.579 in OS (Table 3). There were 52.9~54.3% individual-locus combinations altered their methylation status from CK to OS in the four groups (S6 Table), resulting in the separation of CK and OS samples along the first axis (S3 Fig).

**Table 3:** Percentage of different methylation types, the methylation level, the percentage of polymorphic loci (*PLP*), and the Shannon’s information index (*H’*) of different groups in normal condition (CK) and osmotic stress (OS).

Condition	Group	N	11(%)	01(%)	10(%)	00(%)	Methylation level (%)	*PLP* (%)	*H’*
CK	*Japonica*-upland	71	44.8	20.8	18.2	13.0	54.4	93.5	0.540
	*Japonica*-lowland	59	40.9	23.3	19.1	13.5	58.4	91.4	0.538
	*Indica*-upland	24	43.5	21.2	17.8	13.2	55.4	81.2	0.541
	*Indica*-lowland	26	41.9	22.8	17.6	12.6	56.8	79.7	0.532
	Overall	180	42.7	22.0	18.4	13.2	56.1	100	0.586
OS	Japonica-upland	71	41.3	21.8	16.1	17.3	57.9	93.7	0.573
	Japonica-lowland	59	43.3	21.4	15.8	16.4	56.1	87.4	0.528
	Indica-upland	24	38.5	19.3	19.2	16.6	59.5	81.0	0.558
	Indica-lowland	26	36.9	21.2	17.8	17.6	60.8	84.4	0.579
	Overall	180	40.8	21.2	16.7	17.0	57.9	100	0.594

### Epigenetic divergence between upland and lowland rice in CK and OS conditions

**Fig 1:**
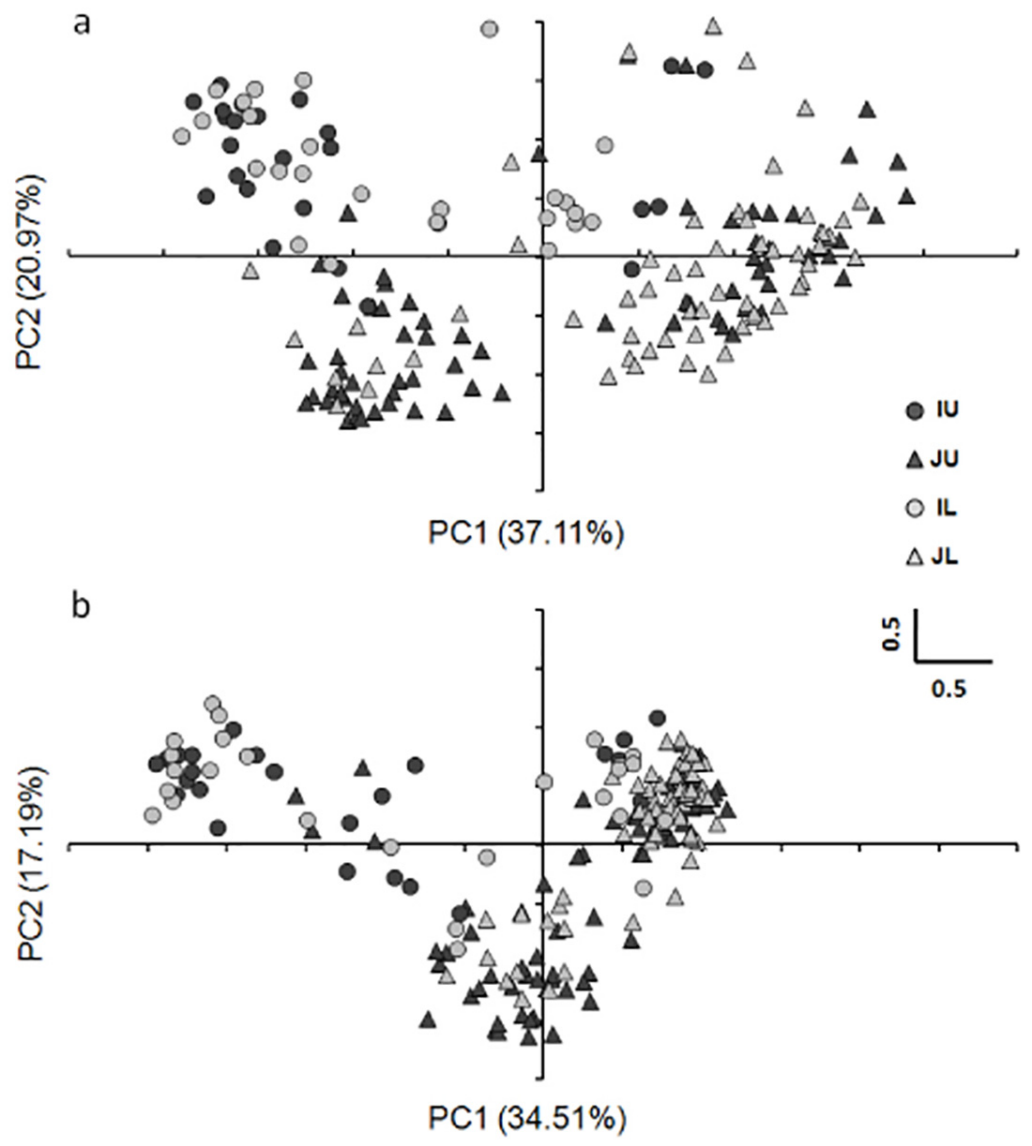
Population structure of rice landraces investigated by Principal Coordinate Analysis in the CK (a) and OS (b) conditions using total sub-epiloci. IU: *indica*-upland; IL: *indica*-lowland; JU: *japonica*-upland; JL: *japonica*-lowland.

The result of PCoA using total sub-epiloci of CK separated *japonica* and *indica* subspecies along the first coordinate, as well as some of the *japonica*-upland and *japonica*-lowland landraces (Fig 1a). It was very similar when total sub-epiloci in the OS condition were used (Fig 1b). Noticeably, the distance matrix of the rice landraces constructed from the total sub-epiloci was significantly correlated with the matrices respectively constructed from four types of sub-epiloci separately both in CK (S4 Fig) and OS (S5 Fig) with similar *R* squares.

Generally, very low levels of epigenetic variance were detected between upland and lowland rice. For example, the AMOVA based ΦST was 2.7%between *japonica*-upland and *japonica*-lowland rice, while it was only 1.9% between *indica*-upland and *indica*-lowland rice (Table 4) in CK condition. The ΦST became even lower when rice encountering osmotic stress. The epigenetic divergence (ΦST) was much lower than genetic divergence (ΦCT) calculated from the SSR (Table 4).

**Table 4:** ΦST among different populations, groups, and ecotypes calculated by the hierarchical analysis of molecular variance using epigenetic data.

Groups	ΦST (ΦCT)	*P* value
MSAP CK (1198)		
All population (n = 9)	0.112	0.0001
Subspecies (n = 2)	0.103	0.0001
Ecotypes (n = 2)	0.016	0.0002
J-ecotypes (n = 2)	0.027	0.0001
I-ecotypes (n = 2)	0.019	0.0133
MSAP OS (1188)		
All population (n = 9)	0.086	0.0001
Subspecies (n = 2)	0.092	0.0001
Ecotypes (n = 2)	0.012	0.0002
J-ecotypes (n = 2)	0.024	0.0001
I-ecotypes (n = 2)	0.005	0.1790
SSR (47)		
All population (n = 9)	0.357	0.0001
Subspecies (n = 2)	0.454	0.0001
Ecotypes (n = 2)	0.027	0.0021
J-ecotypes (n = 2)	0.074	0.0001
I-ecotypes (n = 2)	0.035	0.0002

**Fig 2:**
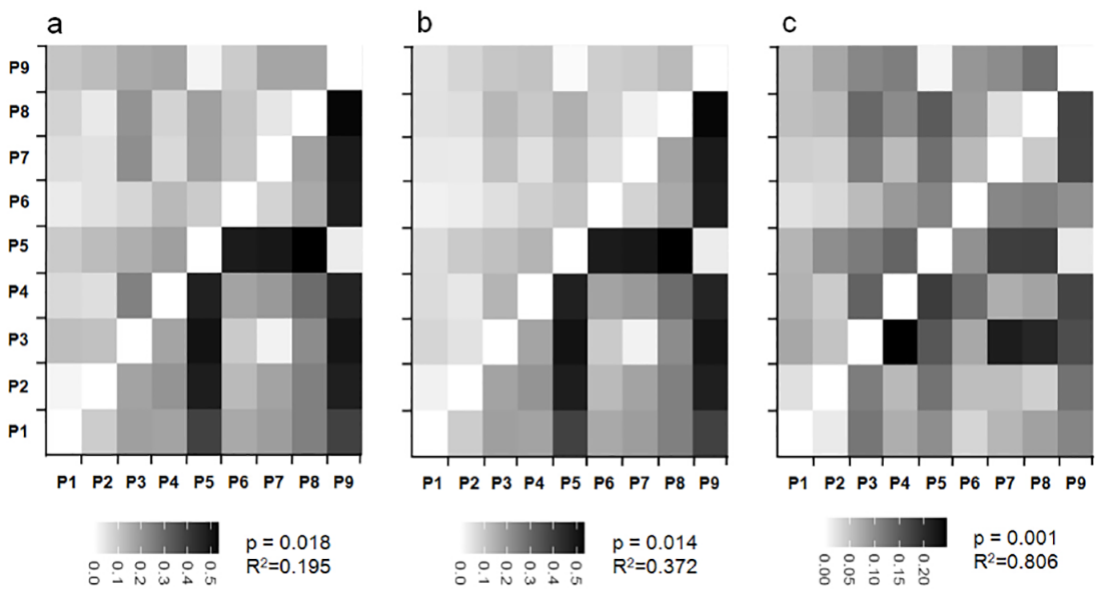
Pairwise ΦST and ΦCT among the 9 populations in CK and OS conditions and their correlations. a) Correlation between pairwise ΦST of epigenetic data in CK (upper) and pairwise ΦCT of genetic SSR data (below). b) Correlation between pairwise ΦST of epigenetic data in OS (upper) and pairwise ΦCT of genetic SSR data (below). c) Correlation between pairwise ΦST of epigenetic data in OS (upper) and in OS (below). The *p* values and *R* squares indicate the correlations *via* Mantel tests.

The matrices of pairewise ΦST from total sub-epiloci among rice populations in CK (Fig 2a) and OS (Fig 2b) were poorly correlated with that from SSR data, demonstrating obvious differences between epigenetic and genetic structures. For example, the ΦCT between Pop3 and Pop7from Hebei province was the lowest, while the ΦST between the two populations was the highest (Fig 2). Additionally, the matrix of pairwise ΦSTs in CK was highly correlated with that in OS (Fig 2c).

### Adaptive divergence between upland and lowland rice on highly divergent epiloci

In *japonica* subspecies, 94 sub-epiloci were detected as HDE-CK (Panel A in S6 Fig) in while 82 sub-epiloci were detected as HDE-OS (Panel C in S6 Fig). In *indic*a subspecies, 77 sub-epiloci were detected as HDE-CK (Panel B in S6 Fig) while 89 sub-epiloci were detected as HDE-OS (Panel D in S6 Fig). Noticeably, there were only few HDE shared in *japonica* and *indica* subspecies (S7 Fig). Similarly, the highly divergent epiloci in CK was seldom overlapped with these in OS (S7 Fig).

The ΦST on HDE-CK between upland and lowland ecotypeswere0.137 (p = 0.0001) in *japonica* and 0.187(p = 0.0001) in *indica*, while the ΦST on neutral loci were 0.012 (p = 0.0009) in *japonica* and 0.002(p = 0.2980) in *indica*. The ΦST on HDE-OS between upland and lowland ecotypes were 0.134 (p = 0.0001) in *japonica* and 0.149 (p = 0.0001) in *indica*, while the ΦST on neutral epiloci were 0.012 (p = 0.0001) in *japonica* and -0.009 (p = 0.9574) in *indica*.

The methylation levels of HDE-CK in upland rice was significantly lower than that in lowland rice in both *japonica* (p = 0.034) and *indica* (p = 0.010) subspecies in CK conditions, while they became almost equal (p>0.05) in OS conditions (Table 5). Methylation levels of neutral epiloci were almost the same in upland and lowland rice both in CK and OS conditions (Table 5). Additionally, the lower methylation level of HDE-CK in upland rice was mainly contributed from epiloci of de-methylation type (S7 Table). These results indicated that the HDE-CK, particularly those of de-methylation type, tended to be at lower methylation levels in upland rice in the CK condition. The DMD from CK to OS was significantly higher on the HDE-OS than it on the neutral loci both in *japonica* (0.262±0.022 vs. 0.147±0.008, p<0.001) and *indica* (0.240±0.026 vs. 0.154±0.009, p<0.001).Additionally, there were 32.8% (38 in 119) HDE-OS oppositely altered between upland and lowland rice on their methylation levels when they encountered the OS stress, while the proportion in neutral epiloci was only 15.4% (64 in 415).

**Table 5:** Methylation levels on highly divergent (HDE) and neutral epilociin normal condition (CK) and osmotic stress (OS).

Group	Type of locus	Methylation level	
		CK	OS
*Japonica*-upland	HDE-CK	63.7±3.0*	66.3±3.4
*Japonica*-lowland	HDE-CK	72.4±2.6*	67.2±3.4
*Japonica*-upland	Neutral	50.7±2.4	52.4±2.5
*Japonica*-lowland	Neutral	52.7±2.4	51.8±2.6
*Indica*-upland	HDE-CK	58.8±3.1*	65.0±3.1
*Indica*-lowland	HDE-CK	69.7±2.7*	69.2±2.8
*Indica*-upland	Neutral	55.7±2.1	57.7±2.2
*Indica*-lowland	Neutral	55.3±2.1	58.3±2.3

The values in **bold** and with ‘‘*” indicated significant differences (p<0.05) between upland and lowland ecotypes by independent *t* test.

### Annotations and expressions of genes at HDE

**Fig 3:**
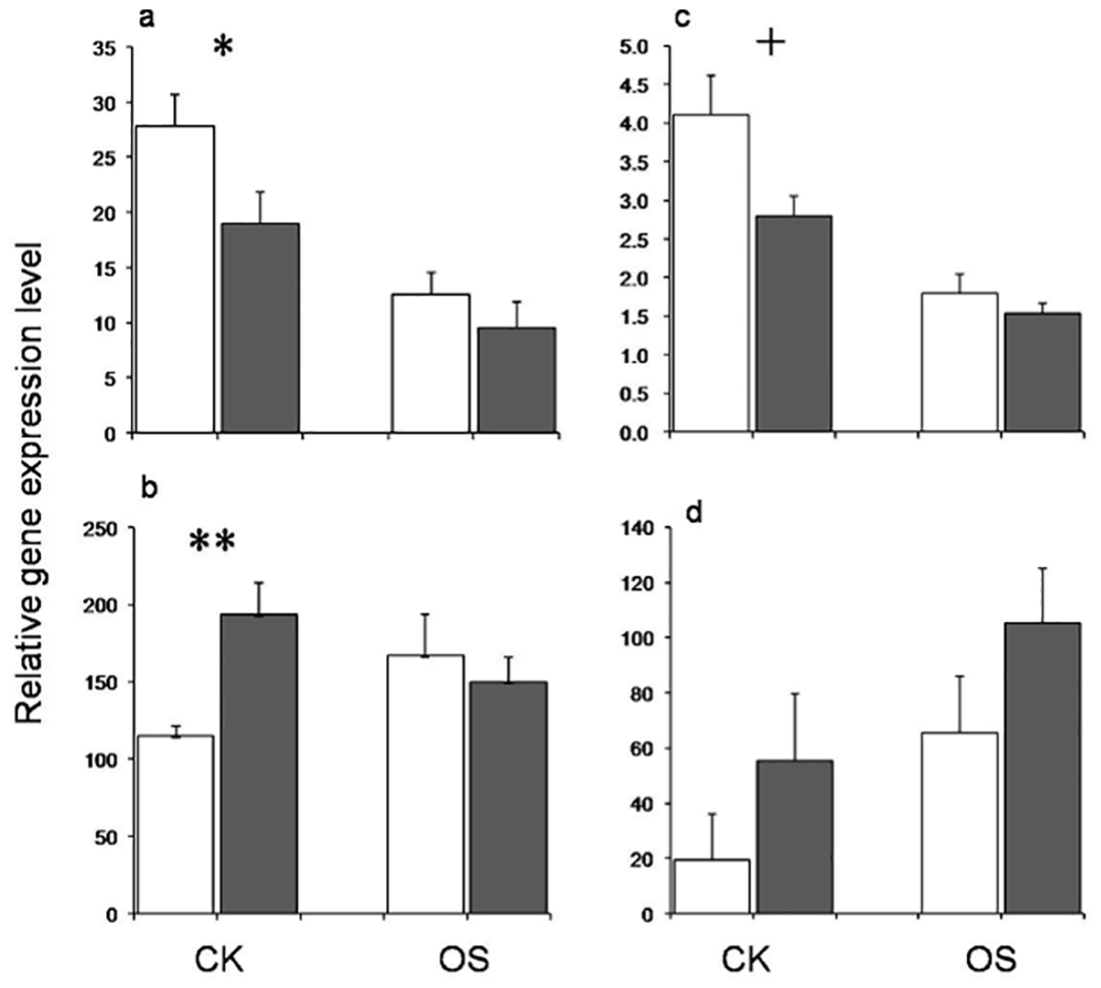
Relative expression levels of genes of highly divergent epiloci in CK and OS conditions. a) LOC_Os05g18604; b) LOC_Os12g44160; c) LOC_Os05g38400; d) LOC_Os02g06160.White bar indicates gene expression in lowland rice while black bar indicates gene expression in upland rice. Error bars indicate standard errors. ‘*’ and ‘+’ indicate the levels of significance of p<0.05 and p<0.10 between upland and lowland rice ecotypes.

There were 118 fragments covering 64 epiloci scored in this study, 62 among which were considered as HDE genes (S3 Table). Three out of four annotated epiloci were successfully validated by the cloning of eluted MSAP bands, indicating a good annotation of MSAP loci using our method (S4 Table). Four HDE genes were selected to test their expressions in upland and lowland rice (Fig 3 and S5 Table). For LOC_Os05g18604 (M093, HDE-CK), the expression level of epigenotype‘1/1’ in upland rice (50% in upland, 22.6% in lowland) was significantly lower [FD (fold difference) = 0.68]than that of epigenotype ‘1/0’ in lowland rice (31.8%in upland, 69.8% in lowland) in CK (Fig 3a). It was down-regulated [FC (fold change) = 0.45in lowland rice while FC = 0.50 in upland rice when encountering osmotic stress. For LOC_Os12g44160 (M312, HDE-CK), the expression level of epigenotype ‘1/0’ in upland rice (49.2% in upland, 17.5% in lowland) was significantly higher (FD = 1.68) than the ‘0/1’ in lowland rice (11.9% in upland, 40.3% in lowland) in CK (Fig 3b).It was up-regulated (FC = 1.45) when encountering osmotic stress in lowland rice. For LOC_Os05g38400 (M280, HDE-CK), the expression level of epigenotype ‘1/1’ in upland rice (25% in upland, 10.9% in lowland) was marginally lower (FD = 0.68) than the epigenotype ‘0/1’ in lowland rice (20.6% in upland, 39.1% in lowland) in CK (Fig 3c). It was down-regulated (FC = 0.44 in lowland rice while FC = 0.55 in upland rice) when encountering osmotic stress. As great variations among landraces, the expression level ofLOC_Os02g06160 (M007, HDE-OS) was apparently higher in upland rice both in CK (FD = 2.84) and OS (FD = 1.61) conditions although with no significances (Fig 3d).It was up-regulated (FC = 3.34 in lowland rice while FC = 1.89 in upland rice) in the response to the osmotic stress. Noticeably, if the expression level of a gene was up-/down-regulated from CK to OS, it was higher/lower in upland rice in the CK condition, suggesting its expression in upland rice was more adaptive to the osmotic stress.

## Discussion

### Great alterations of DNA methylation induced by the osmotic stress

Stress-induced alterations of DNA methylation have been reported to be associated with plant adaptive responses to various stresses [12, 13, 15]. It is proposed that the stress-induced adaptive-associated epigenetic variants can be inherited and receiving nature selections [4, 41]. Therefore, the alterations of DNA methylation in the stress are one of the sources of novel epimutants. In this study, we detected great genome-wide alterations of DNA methylation from CK to OS, covering about ~53% individual-locus combinations by MSAP. This ratio is similar with that detected in rice treated with salt stress (Karan et al. 2012) but much higher than that induced by drought [13]. The great alterations lead to the CK samples separated from the OS samples along the first coordinate. Given the transgenerational inheritance [8, 15, 20], the osmotic-induced genome-wide alterations of DNA methylation in responses to the osmotic stress is the molecular basis of adaptive divergence between upland and lowland rice on their drought-resistances.

### Adaptive epigenetic differentiation between upland and lowland rice on the HDE

Many previous studies focus the epigenetic divergence only in natural environments [26, 29, 40] or normal cultivations [42]. However, the epigenetic variation in the stressed condition also provides informative cues of adaptive epigenetic divergence among plant populations. Therefore, the epigenetic divergence between upland and lowland rice were investigated both in the normal and osmotic conditions. The epigenetic divergence between upland and lowland rice was generally very low in both CK and OS conditions. This is not surprise as the epigenetic divergence always occurs only on adaption-associated epiloci [28, 29, 40]. The HDE-CK and HDE-OS are considered to be evolutionary consequences of rice ecotypes adaption to different agro-ecosystems. As a result, the epigenetic differentiation between these two rice ecotypes on HDE is much higher. The relative poor correlations between the epigenetic and genetic population structures are also detected, similar with many other reports [40]. The explanation is that the epigenetic markers, particularly the epiloci under selections, are more closely aligned with environmental conditions [27–29].

### Evolutionary significance of the HDE when upland and lowland rice adapted to agricultural ecosystems of contrasting soil-water conditions

The methylation status of HDE-CK detected in this study is kept at lower methylation levels in upland rice in normal condition, particularly the epiloci of de-methylation type. This should be meaningful for upland rice adapting to water-limited environments, as low levels of methylation at upstream always associated with higher or quicker responses of gene expression to the stress [43, 44]. The three genes of HDE-CK provide evidences that the ecotype-preferential epigenotypes have impacts on gene expressions and should be therefore associated with stress-resistance in plants. For example, LOC_Os05g18604 (*OsSCP28*) encodes the serine carboxypeptidase which has been reported to be associated with responses against oxidative stress in rice [45]. LOC_Os12g44160 (encoding a putative oxidoreductases) and LOC_Os05g38400 (encoding a FtsH protease) should be also associated with abiotic stress as their functions reported in other species [46–47]. Interestingly, once the expression of a gene is up-/down-regulated when encountering the osmotic stress, its expression was higher/lower in upland rice in the normal condition than that in lowland rice. This result indicated that the upland rice maybe more adaptive to the drought as the expression level of the HDE gene was more close to the level under the stressed conditions.

The HDE-OS tends to oppositely altered from CK to OS between upland and lowland rice, representing different responses of DNA methylation to osmotic stress between upland and lowland rice. However, the gene expression of HDE-OS seems to have the similar pattern in the response to the osmotic stress in upland and lowland rice, reflecting by the LOC_Os02g06160 (encoding a cysteine-rich receptor-like protein kinase). The HDE-OS should be also meaningful for upland rice adapting to water-limited environments as the opposite alterations of DNA methylation in the stress always represents distinguished stress tolerances in divergent plant genotypes [13, 15]. Consequently, the presentation of HDE-OS could also associate to the higher drought-resistance in upland rice, although it requires further evidences.

## Conclusions

The adaptive epigenetic divergence occurs between upland and lowland rice when they adapting to agro-ecosystems of contrasting soil-water conditions, resulting in the highly divergent epiloci (HDE) both in normal (CK) and osmotic stress (OS). The HDE-CK kept at low levels of DNA methylation in upland rice in the normal condition. The HDE-OS tends to alter oppositely between upland and lowland rice, representing distinguished drought-resistance of upland and lowland rice. The HDE should be the transgenerational inherited epimutaions during upland rice adapting to water-limited environments and its expression level is more adaptive to the osmotic stress in the upland ecotype. The HDE between upland and lowland rice should be one of the reasonable explanations to the higher drought-resistance in upland rice. The underlying epigenetic mechanism by which upland rice has achieved higher drought-resistance and adaptively differentiated from lowland rice is also called the methylation-mediated memory of stress-tolerance [48, 49]. This mechanism has the applicable potential in improving stress-tolerance in rice. Additionally, HDE genes could be associated with drought-resistance and applicable as epigenetic markers in drought-resistance breeding.

## Supporting Information

S1 Fig Examples of resulting chromatograms visualized by the Peakscanner ver. 1.0. The y axis indicates the strength of fluorescent signal while the x axis indicates the molecular weight (bp).
(JPG)

S2 Fig Great variation of methylation levels on the 313 epiloci of different groups in CK and OS conditions. a) *japonica* upland (blue bars) and lowland (red bars) rice in CK; b)*indica* upland (blue bars) and lowland (red bars) rice in CK; c) *japonica* upland (blue bars) and lowland (red bars) rice in OS; d)*indica* upland (blue bars) and lowland (red bars) rice in OS.
(JPG)

S3 Fig Population structures of samples in CK (triangle) and OS (circles) conditions investigated by Principal Coordinate Analysis. Blue, green, yellow, and brown respectively represent *japonica*-upland, *japonica*-lowland, *indica*-upland, and *indica*-lowland rice.
(JPG)

S4 Fig Population structures of CK samples investigated by Principal Coordinate Analysis using separated sub-epiloci of methylation type I (un-methylation) (a), II (hemi-methylation) (b), III (fully-methylation) (C), and IV (hyper-methylation) (d). *R* squares indicate their correlations with total sub-epiloci *via* mantel test.
(JPG)

S5 Fig Population structures of OS samples investigated by Principal Coordinate Analysis using separated sub-epiloci of methylation type I (un-methylation) (a), II (hemi-methylation) (b), III (fully-methylation) (C), and IV (hyper-methylation) (d). R squares indicate their correlations with total sub-epiloci *via* mantel test.
(JPG)

S6 Fig Number of highly divergent sub-epiloci (HDE) detected in this study. a) HDE detected in *japonica* subspecies in CK, b) HDE detected in *indica* subspecies in CK, c) HDE detected in *japonica* subspecies in OS, and d) HDE detected in *indica* subspecies in OS. Purple, yellow, green, and pink colors indicate four types of sub-epiloci (I, II, III, and IV) respectively.
(JPG)

S7 Fig Number of highly divergent sub-epiloci (HDE) shared among subspecies and treatments. Purple, yellow, green, and pink colors indicate the HDE detected respectively in *japonica* in CK, *indica* in CK, *japonica* in OS, and *indica* in OS.
(JPG)

S1 Table Information of primer combinations involved in this study. (DOCX)

S2 Table Matrices of raw data and generated from mixed scoring. (XLSX)

S3 Table Information of sequenced MSAP fragments. (XLS)

S4 Table The MSAP epiloci validated by cloning the eluted MSAP bands. (DOCX)

S5 Table Genes selected in qPCR and their epigenotypes in CK and OS conditions. (XLS)

S6 Table Alteration of methylation types from CK to OS in different rice groups. JU; *japonica* upland; JL: *japonica* lowland; J: *japonica*; IU: *indica* upland; IL: *indica* lowland; I: *indica*.
(DOCX)

S7 Table Methylation levels on De-methylation/Re-methylation types of highly divergent epiloci in normal condition. The values in **bold** and with ‘‘*” indicated significant differences (p<0.05) between upland and lowland ecotypes by independent *t* test.
(DOCX)
