# Supplementary figures and images for: Adaptive Epigenetic Differentiation between Upland and Lowland Rice Ecotypes Revealed by Methylation-Sensitive Amplified Polymorphism
Source: PLoS One. 2016 Jul 5;11(7):e0157810. doi: 10.1371/journal.pone.0157810 (PMC4933381; doi:10.1371/journal.pone.0157810)

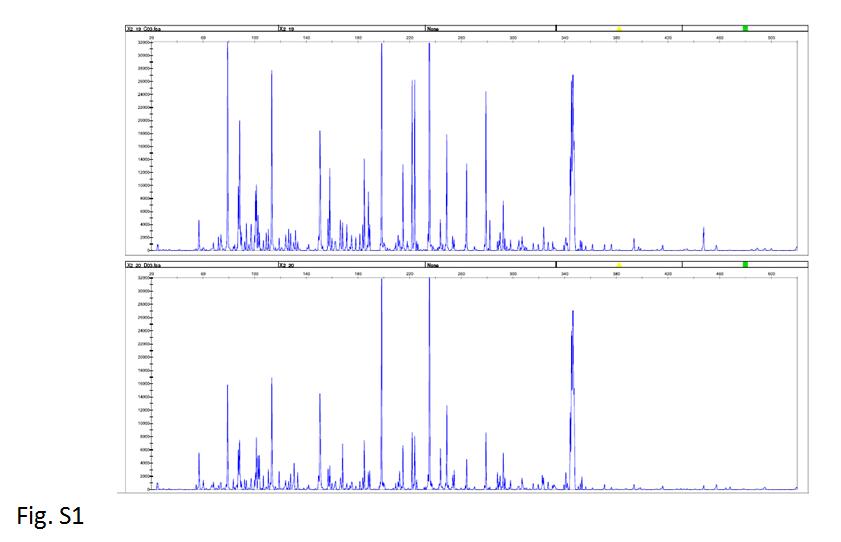

Supplement: S1 Fig — The y axis indicates the strength of fluorescent signal while the x axis indicates the molecular weight (bp). (JPG) [file pone.0157810.s001.jpg]

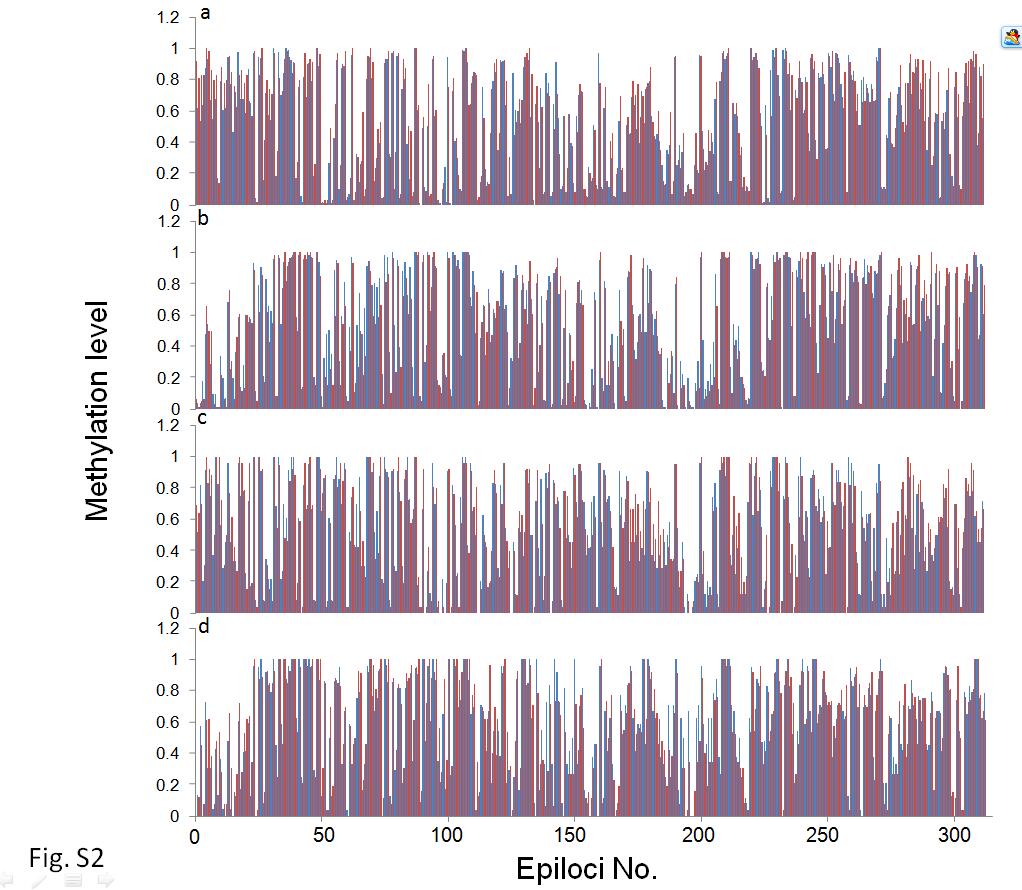

Supplement: S2 Fig — a) japonica upland (blue bars) and lowland (red bars) rice in CK; b)indica upland (blue bars) and lowland (red bars) rice in CK; c) japonica upland (blue bars) and lowland (red bars) rice in OS; d)indica upland (blue bars) and lowland (red bars) rice in OS. (JPG) [file pone.0157810.s002.jpg]

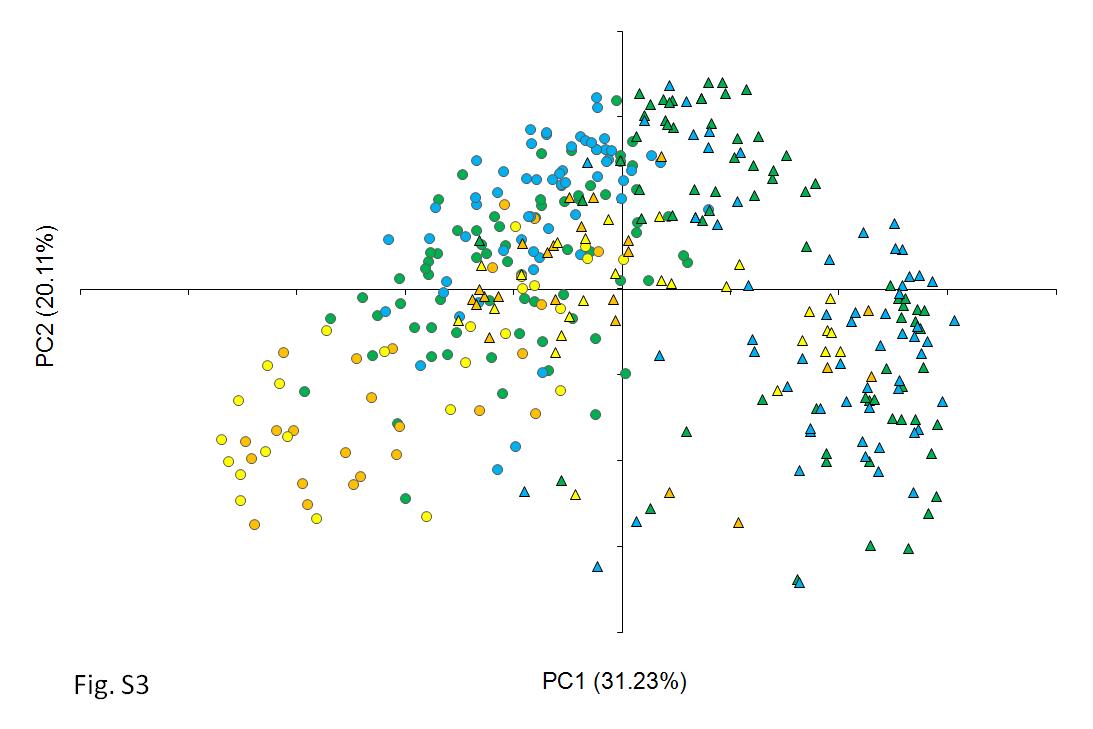

Supplement: S3 Fig — Blue, green, yellow, and brown respectively represent japonica-upland, japonica-lowland, indica-upland, and indica-lowland rice. (JPG) [file pone.0157810.s003.jpg]

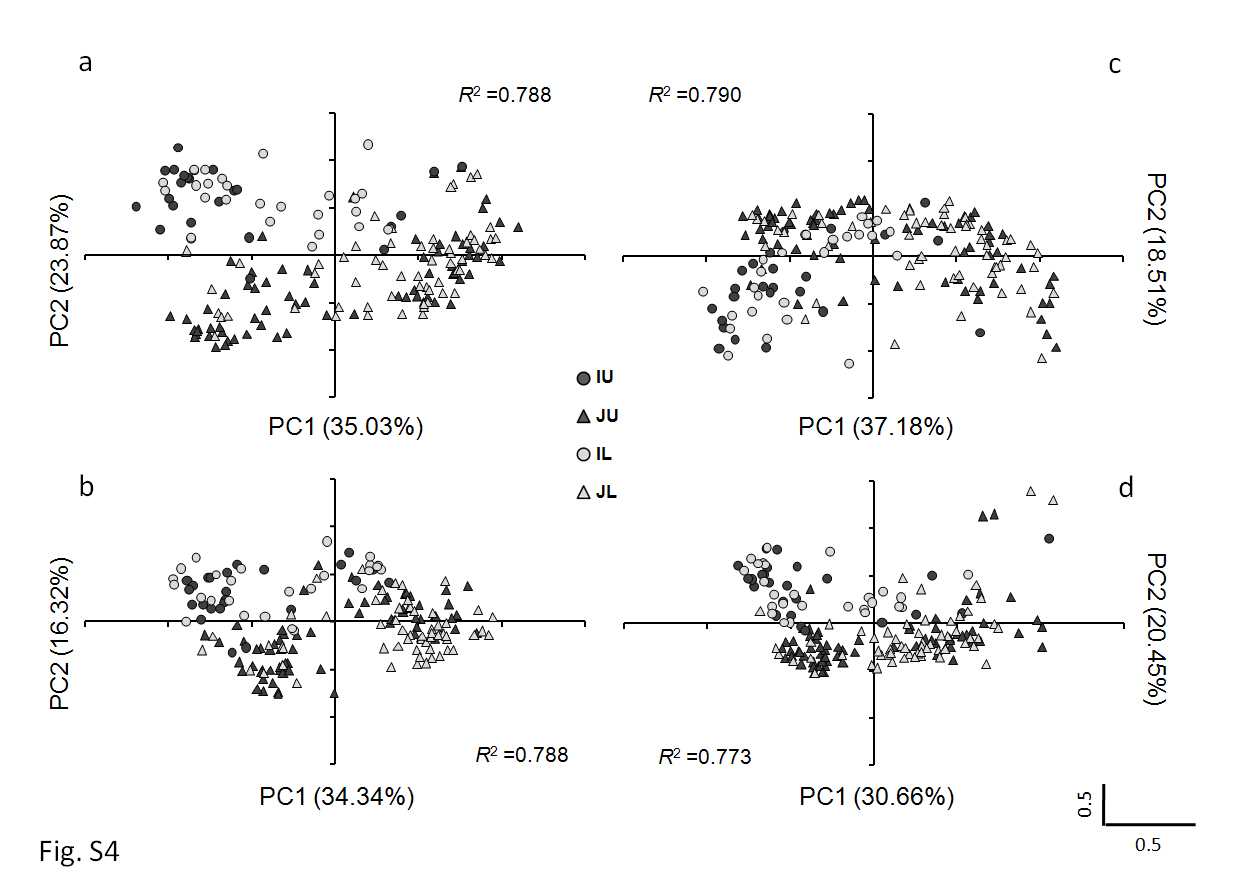

Supplement: S4 Fig — R squares indicate their correlations with total sub-epiloci via mantel test. (JPG) [file pone.0157810.s004.jpg]

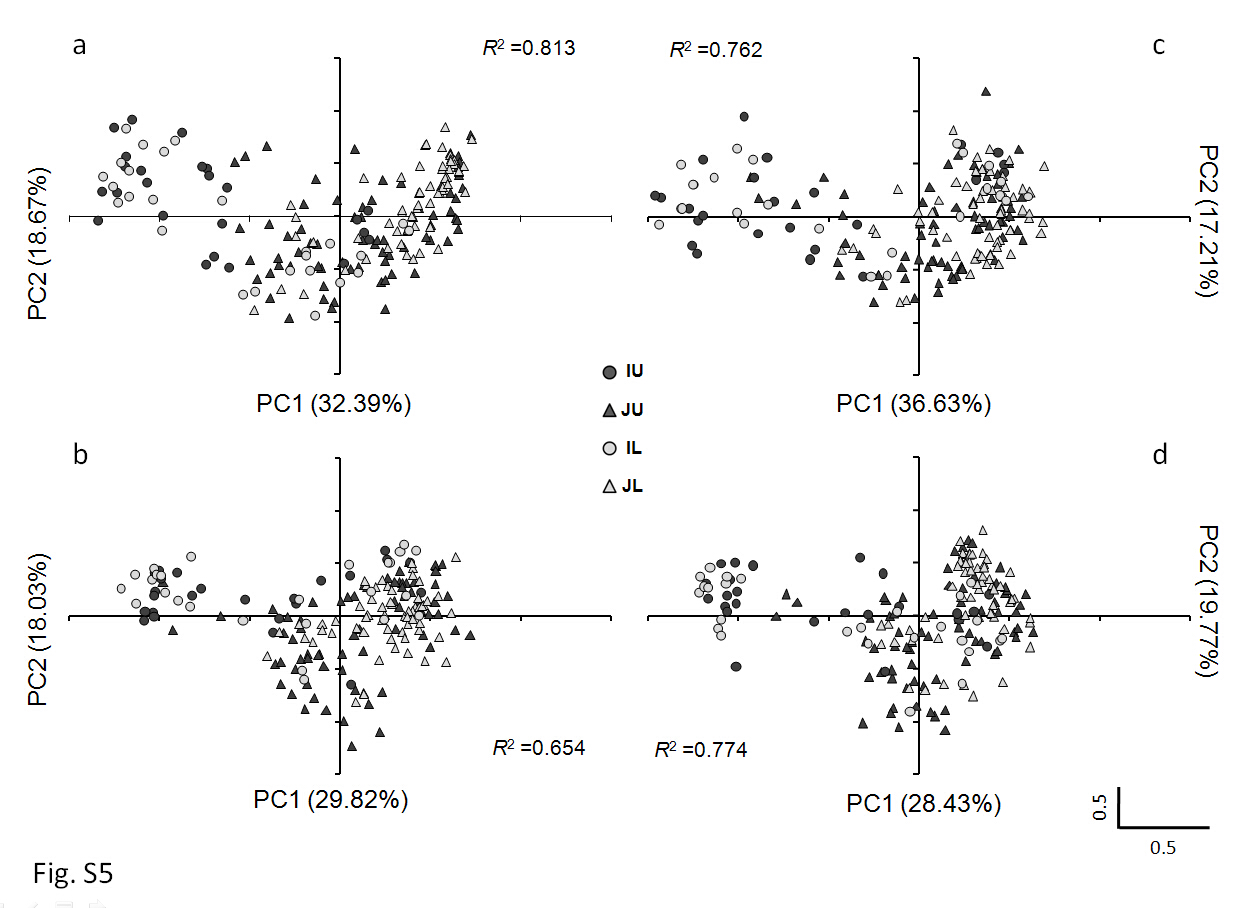

Supplement: S5 Fig — R squares indicate their correlations with total sub-epiloci via mantel test. (JPG) [file pone.0157810.s005.jpg]

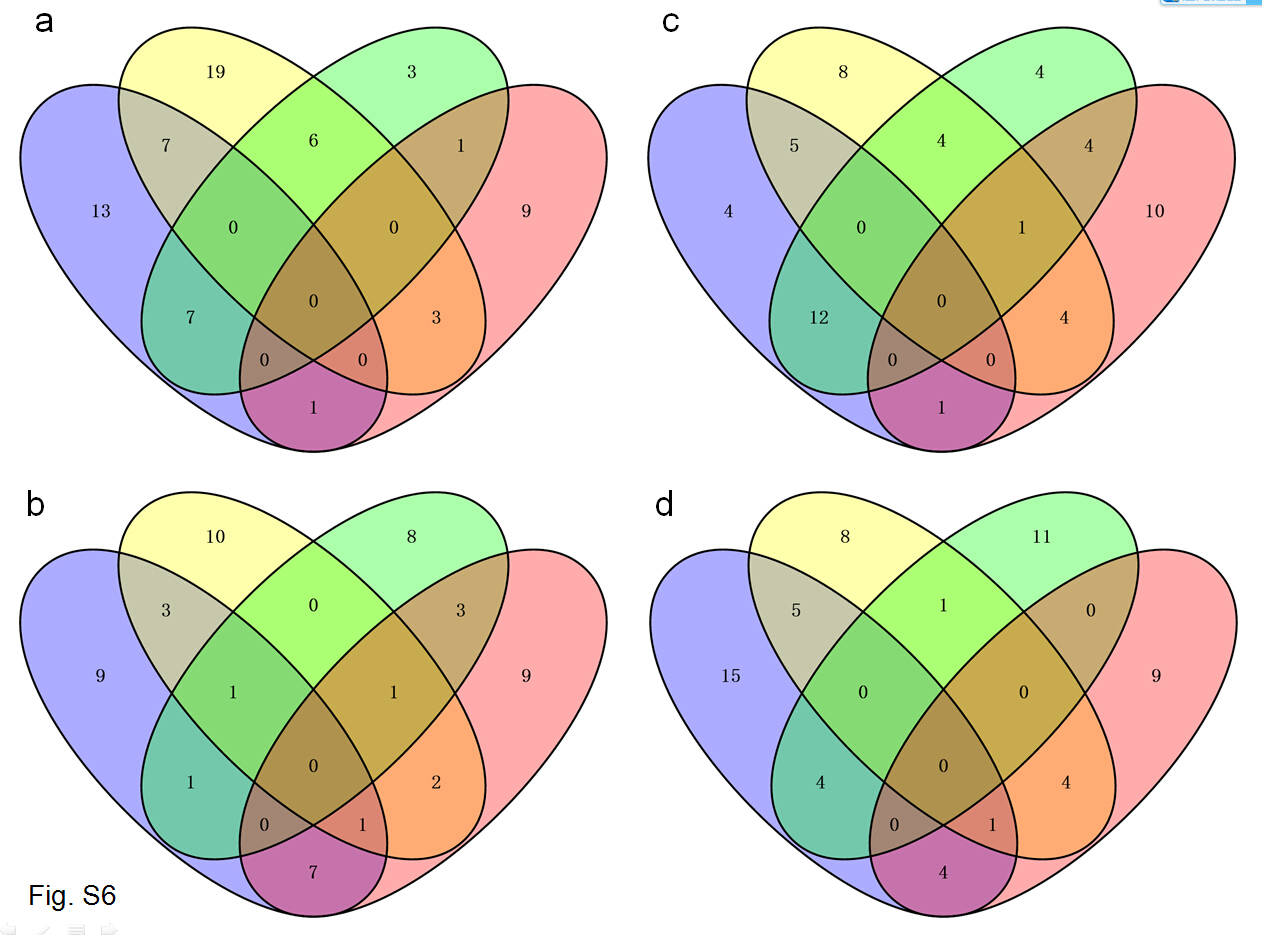

Supplement: S6 Fig — a) HDE detected in japonica subspecies in CK, b) HDE detected in indica subspecies in CK, c) HDE detected in japonica subspecies in OS, and d) HDE detected in indica subspecies in OS. Purple, yellow, green, and pink colors indicate four types of sub-epiloci (I, II, III, and IV) respectively. (JPG) [file pone.0157810.s006.jpg]

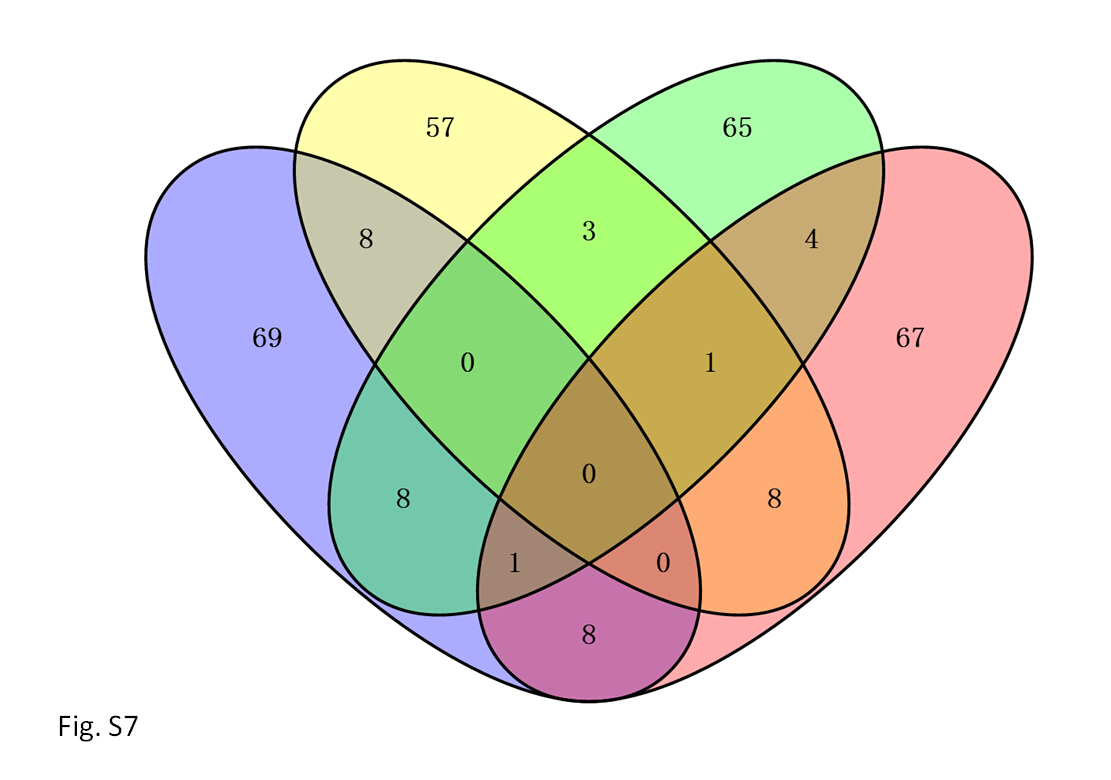

Supplement: S7 Fig — Purple, yellow, green, and pink colors indicate the HDE detected respectively in japonica in CK, indica in CK, japonica in OS, and indica in OS. (JPG) [file pone.0157810.s007.jpg]
