# Supplementary material for: Adaptive Epigenetic Differentiation between Upland and Lowland Rice Ecotypes Revealed by Methylation-Sensitive Amplified Polymorphism
Source: PLoS One. 2016 Jul 5;11(7):e0157810. doi: 10.1371/journal.pone.0157810 (PMC4933381; doi:10.1371/journal.pone.0157810)
Supplement: S1 Table — (DOCX) [file pone.0157810.s008.docx]

**S1 Table** Information of primer combinations involved in this study.

| Primer combination | Forward/ Reverse primer (5’ to 3’) | No. of epiloci | Scoring error (H) | Scoring error (M) |
| --- | --- | --- | --- | --- |
| E03-HM39 | GACTGCGTACCAATTCA**TC**/ GATGAGTCTAGAACGGT**CA** | 20 | 7.70 | 9.09 |
| E06-HM38 | GACTGCGTACCAATTCA**GT**/GATGAGTCTAGAACGGT**GC** | 13 | 4.91 | 10.30 |
| E06-HM313 | GACTGCGTACCAATTCA**GT**/GATGAGTCTAGAACGGT**TA** | 8 | 9.09 | 9.30 |
| E07-HM37 | GACTGCGTACCAATTCA**CA**/GATGAGTCTAGAACGGT**GG** | 8 | 4.03 | 11.36 |
| E07-HM316 | GACTGCGTACCAATTCA**CA**/GATGAGTCTAGAACGGT**TT** | 39 | 8.36 | 11.55 |
| E08-HM34 | GACTGCGTACCAATTCA**CG**/GATGAGTCTAGAACGGT**AT** | 21 | 4.15 | 8.97 |
| E09-HM38 | GACTGCGTACCAATTCA**CT**/GATGAGTCTAGAACGGT**GC** | 25 | 6.42 | 8.97 |
| E10-HM312 | GACTGCGTACCAATTCA**CC**/GATGAGTCTAGAACGGT**CC** | 17 | 7.36 | 7.64 |
| E11-HM311 | GACTGCGTACCAATTCA**TT**/GATGAGTCTAGAACGGT**CG** | 21 | 8.00 | 6.36 |
| E12-HM34 | GACTGCGTACCAATTCA**GG**/GATGAGTCTAGAACGGT**AT** | 13 | 5.85 | 8.30 |
| E12-HM314 | GACTGCGTACCAATTCA**GG**/GATGAGTCTAGAACGGT**AT** | 28 | 6.76 | 4.76 |
| E13-HM35 | GACTGCGTACCAATTCA**AG**/GATGAGTCTAGAACGGT**GA** | 23 | 9.79 | 9.45 |
| E13-HM312 | GACTGCGTACCAATTCA**AG**/GATGAGTCTAGAACGGT**CC** | 12 | 5.45 | 8.75 |
| E14-HM36 | GACTGCGTACCAATTCA**AC**/GATGAGTCTAGAACGGT**GT** | 37 | 9.27 | 5.24 |
| E15-HM33 | GACTGCGTACCAATTCA**AT**/GATGAGTCTAGAACGGT**AC** | 7 | 6.88 | 3.52 |
| E16-HM314 | GACTGCGTACCAATTCA**AA**/GATGAGTCTAGAACGGT**TG** | 21 | 6.94 | 4.33 |
