# Supplementary material for: Adaptive Epigenetic Differentiation between Upland and Lowland Rice Ecotypes Revealed by Methylation-Sensitive Amplified Polymorphism
Source: PLoS One. 2016 Jul 5;11(7):e0157810. doi: 10.1371/journal.pone.0157810 (PMC4933381; doi:10.1371/journal.pone.0157810)
Supplement: S4 Table — (DOCX) [file pone.0157810.s011.docx]

**S4 Table** The MSAP epiloci validated by cloning the eluted MSAP bands.

| MASP epiloci | Molecular weight | Number of clones | Validation |
| --- | --- | --- | --- |
| M102 | 248 bp | 3 | yes |
| M112 | 108 bp | 1 | yes |
| M117 | 164 bp | 1 | yes |
| M124 | 198 bp | 2 | no |
