# Supplementary material for: Adaptive Epigenetic Differentiation between Upland and Lowland Rice Ecotypes Revealed by Methylation-Sensitive Amplified Polymorphism
Source: PLoS One. 2016 Jul 5;11(7):e0157810. doi: 10.1371/journal.pone.0157810 (PMC4933381; doi:10.1371/journal.pone.0157810)
Supplement: S6 Table — JU; japonica upland; JL: japonica lowland; J: japonica; IU: indica upland; IL: indica lowland; I: indica. (DOCX) [file pone.0157810.s013.docx]

**S6 Table** Alteration of methylation types from CK to OS in different rice groups. JU; *japonica* upland; JL: *japonica* lowland; J: *japonica*; IU: *indica* upland; IL: *indica* lowland; I: *indica*

| Groups |  | De-methylation (%) | | | | | Methylation (%) | | | | | | Unchanged (%) | | | | Change rate  (%) |
| --- | --- | --- | --- | --- | --- | --- | --- | --- | --- | --- | --- | --- | --- | --- | --- | --- | --- |
|  | 00_01 | 00_10 | 00_11 | 01_10 | 01_11 | 10_11 | 11_00 | 11_10 | 11_01 | 10_00 | 10_01 | 01_00 | 00_00 | 11_11 | 10_10 | 01_01 |  |
| JU | 4.70 | 2.57 | 2.62 | 1.65 | 4.18 | 7.24 | 4.31 | 7.44 | 5.69 | 4.43 | 2.12 | 5.61 | 4.84 | 28.03 | 4.82 | 9.75 | 52.90 |
| JL | 4.58 | 2.89 | 2.85 | 2.73 | 5.19 | 8.31 | 3.64 | 6.07 | 4.39 | 4.28 | 2.51 | 5.47 | 4.74 | 27.60 | 4.44 | 10.32 | 53.30 |
| J | 4.66 | 2.72 | 2.72 | 2.14 | 4.64 | 7.73 | 3.99 | 6.81 | 5.09 | 4.36 | 2.29 | 5.55 | 4.78 | 27.89 | 4.63 | 10.00 | 53.06 |
| IU | 3.58 | 3.53 | 3.01 | 2.09 | 5.30 | 6.18 | 4.62 | 8.67 | 5.37 | 4.45 | 1.91 | 4.90 | 6.93 | 24.87 | 5.55 | 9.03 | 53.94 |
| IL | 3.97 | 3.08 | 2.98 | 2.16 | 4.83 | 5.97 | 4.79 | 7.88 | 5.62 | 4.48 | 2.18 | 6.00 | 6.61 | 24.03 | 5.22 | 10.19 | 54.34 |
| I | 3.81 | 3.31 | 3.00 | 2.11 | 5.06 | 6.07 | 4.69 | 8.26 | 5.48 | 4.46 | 2.01 | 5.49 | 6.74 | 24.56 | 5.33 | 9.61 | 54.15 |
