# Supplementary material for: Adaptive Epigenetic Differentiation between Upland and Lowland Rice Ecotypes Revealed by Methylation-Sensitive Amplified Polymorphism
Source: PLoS One. 2016 Jul 5;11(7):e0157810. doi: 10.1371/journal.pone.0157810 (PMC4933381; doi:10.1371/journal.pone.0157810)
Supplement: S7 Table — The values in bold and with ‘‘*” indicated significant differences (p<0.05) between upland and lowland ecotypes by independent t test. (DOCX) [file pone.0157810.s014.docx]

**S7 Table** Methylation levels on De-methylation/Re-methylation types of highly divergent epiloci in normal condition. The values in **bold** and with **‘‘*’’** indicated significant differences (p<0.05) between upland and lowland ecotypes by independent *t* test.

| Ecotype | Type of loci | Number | Methylation level | p-value |
| --- | --- | --- | --- | --- |
| JU | De-methylation | 24 | **62.5±4.6** | 0.038 |
| JL |  |  | **75.7±3.6** |  |
| JU | Re-Methylation | 26 | 68.8±4.7 | 0.384 |
| JL |  |  | 74.1±3.6 |  |
| IU | De-methylation | 19 | 71.3±4.8 | 0.112 |
| IL |  |  | 81.6±4.4 |  |
| IU | Re-Methylation | 23 | 51.0±4.9 | 0.243 |
| IL |  |  | 58.3±3.8 |  |
